# Epidemiology and healthcare resource utilization in atopic dermatitis in Colombia: A retrospective analysis of data from the National Health Registry from 2015 to 2020

**DOI:** 10.7705/biomedica.6666

**Published:** 2023-03-30

**Authors:** Ángela María Londoño, Juan Raúl Castro-Ayarza, Amira Kronfly, Diana Camila Buitrago, Daniel Felipe Samacá

**Affiliations:** 1 Departamento de Dermatología, Universidad CES, Medellín, Colombia Universidad CES Universidad CES Medellín Colombia; 2 Departamento de Dermatología, Hospital Universitario Nacional de Colombia, Bogotá, D.C., Colombia Hospital Universitario Nacional de Colombia Bogotá, D.C. Colombia; 3 Abbvie, Bogotá, D.C., Colombia Abbvie Bogotá, D.C. Colombia; 4 IQVIA Colombia, Bogotá, D.C., Colombia IQVIA Bogotá, D.C. Colombia

**Keywords:** Dermatitis, atopic/epidemiology, drug therapy, COVID-19, utilization review, Colombia, dermatitis atópica/epidemiología, tratamiento farmacológico, COVID-19, revisión de utilización de recursos, Colombia

## Abstract

**Introduction:**

Atopic dermatitis, also known as eczema or atopic eczema, is a chronic inflammatory skin disorder characterized by the presence of pruritus accompanied by itching. In Colombia, epidemiological and healthcare resource utilization information regarding this pathology is limited.

**Objective:**

To describe atopic dermatitis epidemiological characteristics and healthcare resource utilization patterns in Colombia.

**Material and methods:**

A retrospective database study using real-world data obtained from the national claims database SISPRO (*Sistema de Información para la Protección Social*) for the 2015-2020 period was carried out. Sociodemographic (age, and health services delivery), epidemiological (incidence, prevalence, and comorbidities), and healthcare resource utilization data were extracted from the SISPRO database.

**Results:**

The epidemiological results showed increased incidence and prevalence of atopic dermatitis in Colombia in the 2018-2019 period compared to 2015-2017. Accordingly, the number of medical consultations (particularly with specialists), the number of procedures, and the number of hospitalizations of patients with atopic dermatitis increased. Topic and systemic corticoids were the most frequently prescribed drugs.

**Conclusions:**

Diagnoses of atopic dermatitis in Colombia increased with a concomitant increase in healthcare resource utilization during 2015-2020, which was possibly slowed down by the arrival of the Covid-19. This study may help physicians gaining a better understanding of the disease, improving atopic dermatitis patient management.

## Resumen

**Introducción.:**

La dermatitis atópica, también conocida como eczema o eczema atópico, es un trastorno inflamatorio crónico de la piel caracterizado por la presencia de prurito acompañado de picor. En Colombia, la información epidemiológica y de utilización de recursos sanitarios sobre esta enfermedad es limitada.

**Objetivo.:**

Describir las características epidemiológicas y los patrones de utilización de recursos sanitarios para la dermatitis atópica en Colombia.

**Material y métodos.:**

Se trata de un estudio retrospectivo en el cual se utilizan datos de la práctica clínica real obtenidos del registro nacional SISPRO (Sistema de Información para la Protección Social) en el período 2015-2020. Se extrajeron datos sociodemográficos (incluida la edad y la prestación de servicios de salud), epidemiológicos (incluidos la incidencia, la prevalencia y las comorbilidades) y los correspondientes a la utilización de los recursos sanitarios.

**Resultados.:**

Los resultados epidemiológicos han demostrado un aumento de la incidencia y prevalencia de la dermatitis atópica en Colombia en el periodo 20182019, en comparación con el periodo 2015-2017. Aumentó el número de consultas médicas (particularmente, con especialistas) de pacientes con dermatitis atópica, el de procedimientos y el de hospitalizaciones. Los corticoides tópicos y sistémicos fueron los medicamentos más prescritos.

**Conclusiones.:**

Los diagnósticos de dermatitis atópica en Colombia aumentaron con un incremento concomitante en la utilización de recursos sanitarios durante 2015-2020, que posiblemente se vio atenuado por la llegada del Covid-19. Este estudio puede ayudar a los médicos a tener un mejor conocimiento de la enfermedad y, por lo tanto, mejorar el tratamiento de los pacientes con dermatitis atópica.

Atopic dermatitis, also known as eczema or atopic eczema, is a chronic inflammatory skin disorder characterized by pruritus accompanied by itching ^1^. Its development is believed to involve complex interactions between genetic and environmental factors ^2^. Atopic dermatitis has been linked with several types of skin barrier dysfunctions related to mutations in skin protein genes, alterations in the immune response, and IgE-mediated hypersensitivity ^3^.

This pathology usually begins during infancy, with a prevalence peak of 15 to 20% in early childhood ^4^^-^^6^. Even though atopic dermatitis often subsides by late childhood in most patients, it can persist into adolescence and even adulthood, with an estimated prevalence ranging from 2 to 5% ^4^^-^^7^. Most patients have mild disease and experience intermittent flare-ups with spontaneous remissions. However, about 40% of adults who suffer atopic dermatitis have a moderate to severe disease ^8^, with symptoms that can significantly impact their quality of life and rarely decrease without treatment ^9^^-^^12^.

Currently, there is no cure for atopic dermatitis and therapies focus on reducing the duration of symptoms to keep the patient in remission and avoid relapse ^1^. Treatments can be topical and/or systemic and aim to control the maintenance of the skin barrier function and modulate the excessive immune response characteristic of the clinical picture ^13^^,^^14^. Topical treatments are the mainstay of eczema therapy, including tar, sulfur, and emollients. However, moderate to severe atopic dermatitis treatment includes other therapeutic approaches that have changed dramatically over the last decades ^15^. Corticosteroids -both topic and systemic- have been used since the 1950s ^16^, whereas other forms of systemic therapy, including cyclosporine, started being used between the 1970s and 1990s ^17^^-^^19^. Dupilumab is, since 2017, the only approved biological therapies for the treatment of moderate to severe atopic dermatitis ^20^, even though multiple biologic therapies have been proposed as treatment alternatives during the last two decades ^21^^,^^22^.

The discomfort caused by atopic dermatitis symptoms, which are not always alleviated due to the limited treatment options, cause a high impact on quality of life, reaching the highest rates among skin diseases ^23^. Moreover, although it is difficult to estimate, atopic dermatitis is associated with a significant impact on healthcare resource utilization, with high associated costs incurred by patients, caregivers, and healthcare systems ^24^^-^^26^.

Due to the characteristics of this disease, understanding the epidemiological characteristics and healthcare resource utilization patterns of atopic dermatitis is of great importance to developing healthcare strategies. In Colombia, few studies have investigated the landscape of atopic dermatitis and additional evidence regarding treatment decisions, potential unmet medical needs, and outcomes are needed ^27^^,^^28^.

This retrospective real-world study aimed to describe the epidemiological characteristics and healthcare resource utilization patterns of atopic dermatitis in the Colombian public healthcare system during the 2015-2020 period using a comprehensive publicly available database collecting information from all healthcare providers in the public healthcare system.

## Material and methods

### Study design and population

This was a retrospective database study employing real-world data to describe the epidemiological and healthcare resource utilization characteristics associated with atopic dermatitis diagnosis in Colombia. Patients from all age groups were included, having at least one registered health claim associated with three ICD-10 codes: L20.0 (Besnier’s prurigo), L20.8 (Other atopic dermatitis), and L20.9 (Atopic dermatitis, unspecified), which are assumed to represent the entire population of atopic dermatitis in the country. Data was retrieved from ISPRO (*Sistema de Información para la Protección Social*), a national claims database, for a 5-year period (2015-2020). The Colombian Ministerio de Salud y Protección Social uses SISPRO to collect healthcare system information, including all healthcare providers in the national public healthcare system (*Sistema General de Seguridad Social en Salud*, SGSSS), covering about 96% of the Colombian population. This information is public and is available for research purposes.

The SISPRO database is structured in independent modules for inpatient, outpatient, and pharmacy data. Due to different financing sources, pharmacy data are distributed across two different modules within the national database: SUF (*Cubo de Gestión de la Demanda*) and MIPRES (*Mi prescripción*). The SUF module includes information regarding medications covered by the national health benefits package. The study was performed in accordance with the principles of the Helsinki Declaration.

### Variables

This study included sociodemographic, epidemiological (incidence and prevalence), clinical, and healthcare resource utilization variables. Sociodemographic variables, including age, residence, and health service provider were considered. Prevalence and incidence were extracted from the number of atopic dermatitis claims per year and the number of new individuals with atopic dermatitis claims per year reported in the registry, respectively. Specifically, incident cases were identified through two filters available in the RIPS module of SISPRO: new confirmed cases and diagnosis impression. Data were additionally stratified by age groups (0-11, 12-17, 1829, 30-59, and above 60 years of age). Clinical variables were concomitant diagnosis (i.e., comorbidities) and was a secondary outcome, extracted from the SUF module, with available data up to 2019.

Healthcare resource utilization information included consultations (number and date of outpatient medical visits related to an atopic dermatitis ICD-10 code), hospitalizations (number of registered hospital stays for a patient with an atopic dermatitis ICD-10 code, length of stay, and hospitalization date), procedures (number of inpatient and outpatient procedures performed on a patient with an atopic dermatitis ICD-10 code; the type and date of the procedure were also extracted), laboratory tests (number of laboratory tests performed on a patient with an atopic dermatitis ICD-10 code; the type and date of the test were also extracted) and medication (units of medication given to a patient with an atopic dermatitis ICD-10 code). The total count of patients receiving medication prescriptions of certain drugs of interest was computed by year and age group, and by the specialty prescribing the medicine. The number of procedures and frequency rate per 1,000 patients with atopic dermatitis in a given year was extracted from the database, and the frequency of hospitalization.

Related or unrelated prescription treatments to the ICD-10 code were included, irrespective of coverage by the national health benefits plan. Information about medications dispensed (coded according to ATC classification and a national classification system) and the amount dispensed was extracted from the SUF module of the SISPRO, collecting PBS-covered medications up to 2019. Dupilumab prescription counts are reported in the non-PBS SISPRO module (MIPRES), which reports information from 2017.

The total population count was extracted from the database of enrollees of the SGSSS and DANE (*Departmento Administrativo Nacional de Estadística*) ^29^.

### Statistical analysis

Gathered data were analyzed descriptively, and each point estimator is presented by a dispersion measure where applicable: continuous variables were described with measures of central tendency (means, medians) and dispersion (standard deviation, range), and categorical variables were expressed as absolute numbers and relative frequencies. Ninety-five percent confidence intervals (95%CI) were calculated, where applicable.

Crude rates were adjusted by the total population for each year and by age group as appropriate. Age-standardized rates were calculated from the number of incident or prevalent cases in a specific age group divided by the total number of people in that group, and then weighted by the proportion each age group contributes to the entire population. Incidence and prevalence rates are presented per 100,000 inhabitants.

## Results

### Incidence and prevalence of atopic dermatitis

**Figure 1:**
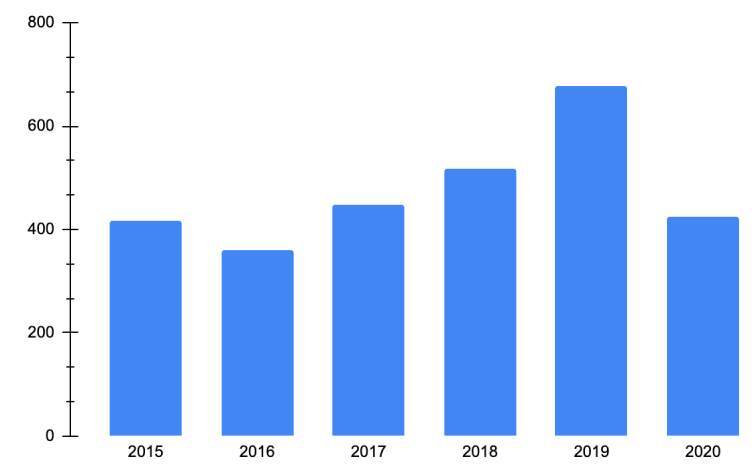
Atopic dermatitis prevalence rate age-standardized adjusted by the total population for each year (per 100,000 inhabitants)

Between 2015 and 2020, we identified 1,090,960 new patients with a diagnosis of atopic dermatitis receiving care (L20.0 - Besnier’s prurigo, L20.8 - Other atopic dermatitis, and L20.9 - Atopic dermatitis, unspecified). Table 1 summarizes crude and age-standardized incidence and prevalence rates for each year. Incidence rates reported between 2015 to 2017 increased, with rates lower than 370 patients per 100,000 inhabitants, compared to the period between 2018 and 2019 with higher incidence rates: 404.5 and 523.7 per 100,000 inhabitants, respectively. Like the incidence rates, prevalence cases showed higher rates in the last years, especially in 2018 and 2019, compared to the period reported between 2015 and 2017, with the highest number of patients with atopic dermatitis reported in 2019 (659.1 per 100,000 inhabitants) (figure 1).

**Table 1:** Epidemiology of atopic dermatitis cases (annual incidence rate per 100,000 inhabitants, prevalent cases, and prevalence rate per 100,000 inhabitants) at the indicated years in the Colombian population

Year	New atopic dermatitis cases	Incidence rate* (per-100,000 inhabitants)	Incidence rate age- standardized** (per 100,000 inhabitants)	Atopic dermatitis prevalent cases	Prevalence rate* (per-100,000 inhabitants)	Prevalence rate age- standardized** (per 100,000 inhabitants)
2015	169,786	361.0	367.0	195,862	416.4	423.4
2016	140,519	301.3	301.2	167,927	360.1	359.1
2017	173,556	371.1	367.7	209,176	447.3	441.9
2018	193,610	410.4	404.5	243,747	516.7	506.5
2019	256,390	534.6	523.7		324,769	677.1
2020	157,099	316.8	317.2		210,256	424.0

* Adjusted by the total population for each year

** Age-standardized incidence is calculated from the number of new cases in a specific age group divided by the total number of people in that group and then weighted by the proportion each age group contributes to the entire population.

**Figure 2:**
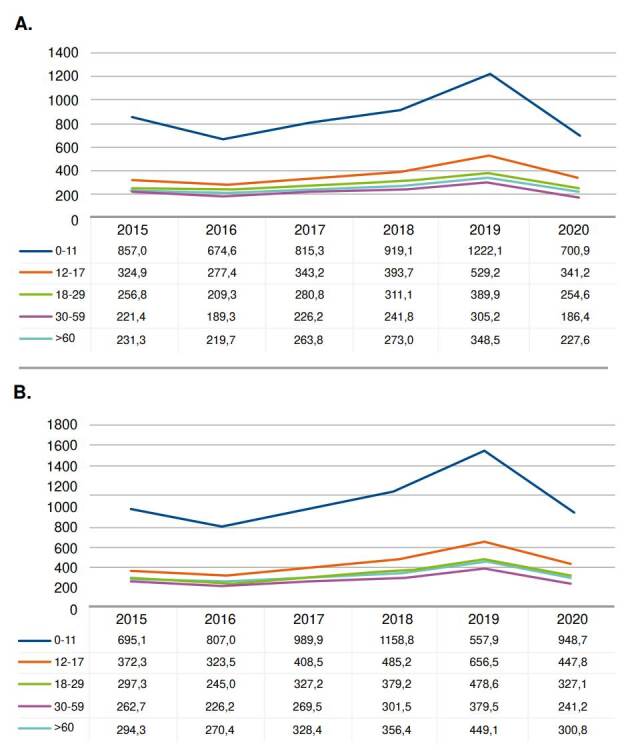
Annual incidence and prevalence rate for atopic dermatitis in the Colombian population by age group

Incidence and prevalence rates by age group are shown in figure 2. The patients with atopic dermatitis who received healthcare between 2015 and 2020 in higher numbers were children of 0-11 years of age, followed by adolescents between 12 and 17 years, and by patients between 30 and 59 years of age and those between 18 and 29 years (figure 2). Adolescents (12 to 18 years old) and older adults (>60) represented a smaller number of cases attended annually during 2015-2020.

### Comorbidities of patients with atopic dermatitis

Supplementary table 1 shows the frequency and distribution of atopic dermatitis patients with concomitant diagnoses. The most common secondary diagnosis was skin and subcutaneous tissue diseases, followed by diagnoses related to factors influencing health status and contact with health services. Skin pathologies had the highest percentage, followed by potential health hazards related to communicable diseases, socioeconomic and psychosocial circumstances, family and personal history, certain conditions influencing health status, and persons encountering health services for examination and investigation, in circumstances related to reproduction or specific procedures and health care.

Data on secondary diagnoses according to the age group of the atopic dermatitis patients identified for each year are summarized in supplementary table 2. The age group with the highest number of atopic dermatitis patients with a secondary diagnosis reported between 2015 and 2019 were children between 0 to 11 years old, followed by adults between 30 and 59 years old, which corresponds to the groups with the highest incidence.

**Table 2:** Number of patients with consultations related to atopic dermatitis classified according to primary care physician and specialist each year in the Colombian population

Consultation type	2015	2016	2017	2018	2019	2020
First-time consultation with primary care physician	92,034	77,875	88,563	95,603	121,158	72,594
Control or follow-up consultation with primary care physician	45,397	37,576	58,480	72,679	103,235	61,137
First-time consultation by a specialist with a dermatologist	7	2,210	10,531	18,247	26,835	18,087
Control or follow-up consultation with a dermatologist	12	620	2,871	4,347	7,171	5,571
Consultation (second opinion) with a dermatologist	-	3	34	70	81	66
First-time consultation by a specialist with an allergist	-	496	1,167	2,166	3,340	2,401
Control or follow-up consultation with an allergist	5	7	148	466	1,225	905
Consultation (second opinion) with an allergist	-	-	3	3	8	3
Total	135,817	116,933	156,209	185,613	251,122	153,532

### Healthcare resource utilization

This study analyzed healthcare resource utilization for patients diagnosed with atopic dermatitis in the SISPRO database. This included the number of consultations per year, the frequency and rate of medication prescriptions for atopic dermatitis treatment, and the frequency of procedures and tests such as phototherapy, skin biopsies, and IgE levels.

### Consultations’ patterns

This study evaluated the frequency of visits for patients with atopic dermatitis to primary care physicians and specialists, including dermatologists and allergists. Table 2 shows the number of patients who attended medical consultations each year of the study, including first-time and follow-up visits by specialty.

There was an increase in the number of atopic dermatitis patients seeking healthcare between 2018 and 2019, reflected in both first-time and followup consultations with primary care physicians and specialists. The absolute increase in primary care physician consultations between 2018 and 2019 was close to 30%, while the absolute increase in specialists was greater than 40%, indicating higher demand for specialty care such as dermatology and allergology. However, a low percentage of patients received specialist care, whether for the first time or follow-up.

### Hospitalization patterns

**Figure 3:**
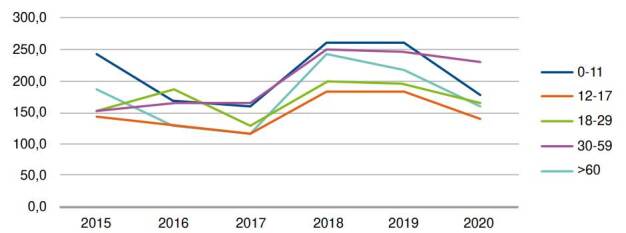
Hospitalization rate of patients (per 100,000) admitted with atopic dermatitis as primary diagnosis

Hospitalization rates for atopic dermatitis increased in 2018 and 2019 but decreased moderately in 2020 (table 3 and figure 3). The highest hospitalization rates per 100,000 atopic dermatitis patients were found in patients between 0 and 11 years of age and adults between 30 and 59 years of age in 2018 and 2019. However, older adults had the highest peak between 2017 and 2018, with 117.8 and 243.7 hospitalized patients per 100,000 atopic dermatitis patients respectively in 2017 and 2018. Despite the differences in hospitalization rates among the different age groups, the overall rates were similar, suggesting that the causes of hospitalization were generally related to the severity of the disease across all age groups.

**Table 3:** Hospitalization rate of patients admitted with atopic dermatitis as primary diagnosis per 100,000 atopic dermatitis patients

Age group (years)	2015	2016	2017	2018	2019	2020
0-11	245.9	169.3	159.1	261.5	263.1	179.7
12-17	146.4	132.3	115.6	182.8	185.1	142.7
18-29	152.6	187.4	129.3	200.7	194.7	164.3
30-59	152.6	164.4	165.0	250.6	246.5	230.8
>60	186.1	129.6	117.8	243.7	218.9	161.1
Total	196.9	163.5	148.0	240.2	237.4	182.3

### Drug utilization

The prescription patterns for atopic dermatitis revealed that topical corticosteroids were the most used treatment, with a rate of 35.76 patients per 100 atopic dermatitis patients in 2016 (table 4). However, the prescription rate for these drugs decreased significantly in 2018 to 15.5 patients per 100 atopic dermatitis patients and remained low in subsequent years. The second most frequently prescribed treatment was systemic corticosteroids, but their prescription rate also decreased significantly from 27.06 per 100 atopic dermatitis patients in 2015 to no more than five per 100 atopic dermatitis patients. Additionally, the prescription rate for cyclosporine increased from 0.07 to 0.19 patients per 100 atopic dermatitis patients between 2018 and 2019. Phototherapy, which is considered a procedure, was not included in this analysis, but its ordering rate was found to be higher than that of cyclosporine and other non-steroidal immunomodulators, indicating that it is a commonly used first-line prescription therapy.

**Table 4:** Percentages of patients with a registered medication prescription per 100 atopic dermatitis patients by study year

	2015	2016	2017	2018	2019	2020
Dupilumab	-	-	-	-	0.19	0.48
Cyclosporine	0.04	0.06	0.06	0.07	0.19	-
Systemic corticosteroids	27.06	4.60	4.11	3.58	2.46	-
Topical corticosteroids	33.03	35.76	29.96	15.50	34.37	-
Methotrexate	0.06	0.04	0.04	0.03	0.03	-
Azathioprine	0.04	0.07	0.07	0.06	0.03	-
Mycophenolate mofetil	0.00	0.00	0.00	0.00	0.00	-

Table 5 summarizes the number of patients with atopic dermatitis who received dupilumab prescriptions between 2019 and 2020, the total number of syringes prescribed during these years, and the average number of syringes prescribed per patient. It is important to note that the number of syringes prescribed per patient may vary according to the treating physician’s criteria.

**Table 5:** Number of dupilumab syringes per patient by age group and by year of study

Age group (years)	2019			2020		
	Patients*	Syringes	Syringes per patient	Patients*	Syringes	Syringes per patient
0-11				8	68	8.5
12-17	22	302	13.7	32	499	15.6
18-29	248	4,298	17.3	432	8,972	20.8
30-59	259	4,770	18.4	390	8,453	21.7
>60	40	558	14.0	66	1,248	18.9
Total	569	9,928	17.4	928	19,240	20.7

* Patients with atopic dermatitis-related diagnosis and prescription of dupilumab

Regarding dupilumab prescriptions from 2019 to 2020, the results indicate a rise in the number of patients with atopic dermatitis who received them (Supplementary table 3). Dermatologists and allergist specialists were the primary providers of these prescriptions, followed by primary care physicians.

### Procedures and test prescription patterns

The frequency of the main procedures and laboratory tests related to a diagnosis of atopic dermatitis were calculated by comparing the number of procedures or tests each year to the total number of atopic dermatitis patients in the same year. The rates varied significantly over the years, particularly for skin biopsies (0.71-1.73 per 100 patients per year). However, the overall trend showed an increase in the frequency rates from 2015 to 2020 for phototherapies, skin biopsies, and IgE tests, from 0.18 to 0.74, 1.05 to 1.73, and 4.38 to 6.95 respectively.

## Discussion

The present study is the first investigation aimed at estimating atopic dermatitis epidemiology and healthcare resource utilization in Colombia through a retrospective analysis of the national claims database SISPRO. The epidemiological results showed an increase in the incidence and prevalence of atopic dermatitis in Colombia in the 2015-2017 period compared to the 2018-2019 period. Regarding healthcare resource utilization, consultation and hospitalization patterns increased during 2018-2019, and topic and systemic corticoids appeared as the most frequently prescribed drugs. Unexpectedly, skin and subcutaneous tissue diseases were the most common comorbidity.

Regarding the incidence, this study reported an inferior value (0.360.67%) compared to previous published works on other countries (2-5%) ^4^^-^^7^. This discrepancy may be due to the different methodology used, in this case utilizing a national claims database registry, which may result in lower diagnosis of atopic dermatitis.

The epidemiological information showed an increase in the incidence rate for 2015-2017 compared to 2019, with incidence rates up to 523.7 per 100,000 inhabitants, indicating a substantial increase in the number of patients who received healthcare and had a new diagnosis of atopic dermatitis. The highest incidence rates were found in the subgroups of children and adolescents, consistent with previous studies on atopic dermatitis showing that approximately 60% of patients develop the disease in the first year of life and 90% within the first five years ^4^^-^^6^. Likewise, almost 20% of children with atopic dermatitis will have persisting symptoms of the disease before two years, and only 16.8% of adults with atopic dermatitis experience onset after adolescence, according to several studies ^2^^,^^4^^-^^6^.

Like incidence rates, prevalence rates raised, showing a consistent increase in the individuals who received healthcare between 2015 and 2020, especially for 2019. In this regard, the most prevalent atopic dermatitis cases in 2019 belonged to the group of children (0-11 years of age) and accordingly, was the age group with most medical visits. These results are consistent with other observational studies showing that, regardless of disease severity, participants sought out their healthcare provider less frequently as they aged ^2^^,^^30^. This behavior raises the need to strengthen the strategies to guarantee the continuous treatment of patients of older ages. Furthermore, the observed increased atopic dermatitis prevalence and incidence rates may result from an improved registry, due to the mandatory reporting.

The epidemiological data for atopic dermatitis collected in this study is consistent with the ISAAC study, the most extensive investigation of the disease with close to two million children included in 100 countries ^31^. This study showed that, although atopic dermatitis has been stable in countries such as the United Kingdom and New Zealand, its prevalence has increased in Latin-American countries, particularly for young children of 6 and 7 years of age ^31^. In Colombia, the TECCEMA study ^27^ focused on children, described how the onset of the disease occurred in 47% of cases before 2 years of age, followed by 37% in children between 3 to 5 years, and 16% after 5 years. These data are consistent with the findings of this study. The increased incidence in 2019 and 2020 may be due to the increased number of people who received healthcare and had a new diagnosis of atopic dermatitis, likely reflecting the response to the awareness campaigns carried out when dupilumab was approved as a new drug for the management of the disease.

Even though studies on healthcare resource utilization in atopic dermatitis are scarce, a study conducted in the United States in 2013 through the US National Health and Wellness Survey, with 75,000 responders nationwide, found that, compared to non-atopic dermatitis patients, patients with atopic dermatitis used significantly more healthcare resources, particularly emergency visits and hospitalizations, which were more than twice that of non-atopic dermatitis controls ^25^.

Although our study did not compare atopic dermatitis patients with healthy controls, we identified an increase in the number of patients who received healthcare between 2018 and 2019, with up to 251,122 patients reporting consultations in 2019. Furthermore, this study results show that most of atopic dermatitis patients are managed in primary care, indicating that probably only the most severe ones are referred to the dermatologist. Therefore, it would be necessary to reinforce the education and training of primary care professionals on atopic dermatitis, so that they can offer the best health services to these patients.

Our results showed that topical and systemic corticosteroids were the most frequent treatments prescribed in this population. These data indicate the follow-up of the clinical guidelines’ recommendations by the clinicians and is in agreement with other studies ^32^^-^^34^.

Other treatments such as cyclosporine and dupilumab were not among the most prescribed options, but their frequency rate increased in the last years. This result might be related to an increased proportion of moderate- severe atopic dermatitis patients or an increased failure of topical or systemic treatments.

However, the lack of stratification of the epidemiological information by disease severity and the unavailability of information regarding previous treatments’ outcomes precluded further analysis to confirm these possibilities. The proportional increases in moderate-severe atopic dermatitis patients and the treatment failure may explain the increased number of biopsies performed in recent years. In this regard, the increased number of biopsies may reflect the need for diagnostic confirmation before starting high-cost treatments such as biological therapies as indicated in the atopic dermatitis management Colombian guidelines ^32^. Unlike corticosteroids, the prescription of mycophenolate was infrequent. Perhaps, its consideration as a treatment for atopic dermatitis in the guidelines could be reviewed ^32^, first because there are better alternatives, as indicated by clinicians’ prescribing patterns, and secondly, due to the lack of (*Instituto Nacional de Vigilancia de Medicamentos y Alimentos*, INVIMA) approval for its prescription.

The hospitalization patterns for atopic dermatitis patients showed increased hospitalization rates between 2018 and 2019, consistent with the epidemiological findings. Additionally, a moderate decrease was identified in the number of patients hospitalized in 2020. One possible explanation of these results could be related to the impact of the COVID-19 emergency on health services since March, 2020. To respond to the global health emergency, health providers allocated the available resources to prevent and treat COVID-19 and neglected the care of the chronic conditions ^35^^,^^36^, likely explaining the decreased healthcare resource utilization for patients with atopic dermatitis in Colombia identified in this study. In this regard, dermatology consultations were the third most affected service during the pandemic in the United States, with a 73% and 37% reduction of medical visits and a 55% and 28% reduction in allergists’ visits by April and May, 2020, respectively ^37^.

Similarly, another report showed that the hospital admissions dropped to 69.2% of predicted admissions during the week ending April 4, 2020, with a second decline again in November, 2020, showing that people once again were delaying or forgoing healthcare due to the emergency or the hospital capacity, delaying treatment of non-COVID-19 conditions ^38^. Moreover, this report shows that admissions for patients age 65 and older were 53.4-63.0% of predicted levels in April 2020, compared to 68.6-75.1% of predicted levels for younger patients ^38^, suggesting that older patients with a higher risk of severe illness or death due to COVID-19 were more hesitant than younger patients to enter a hospital if not necessary. This is consistent with our findings for hospitalized older patients with atopic dermatitis during 2019 and 2020 and could explain the results of this study regarding hospitalizations. Before the pandemic, the hospitalized rate for patients older than 60 years old was 218.9 per 100,000 atopic dermatitis patients, while in 2020, the same group reported a hospitalized rate of 161.1 per 100,000 atopic dermatitis patients, showing a higher decrease than the observed in younger patients.

Some limitations for this study should be considered. First, the databases used in this study rely on administrative claims data for clinical detail. Health databases are subject to data coding limitations, data entry errors, and incomplete or inconsistent information ^39^^,^^40^. Nevertheless, they are also a good data source for epidemiological studies since they collect large amounts of data with quality controls that otherwise would be very difficult to obtain. Moreover, there are precedents with the use of SISPRO in the literature ^41^^-^^43^.

In addition to the limitations of health claims databases, this study may be underestimating the economic burden of atopic dermatitis because it only considered direct healthcare resource utilization, and excluded the indirect consequences of the disease, such as absenteeism from work, limitation in physical activities, and decreased productivity. Furthermore, the significant decrease in the quality of life of patients living with atopic dermatitis was not considered and is an important aspect of patients’ care ^2^. For this reason, other consequences associated with atopic dermatitis in Colombia should be considered in future studies from the healthcare systems and patients’ perspectives.

Another possible limitation of the study is the possible changes that have occurred in the SISPRO registry during the study period with the mandatory registry of atopic dermatitis cases, impacting on the reported incidence and prevalence rates.

In conclusion, to our knowledge, this is the first study to report the epidemiological information and healthcare resource utilization for atopic dermatitis in Colombia and brings together a large amount of data that is useful to increase the body of knowledge about the disease. The number of medical consultations (particularly with specialists), the number of procedures, and the number of hospitalizations of patients with atopic dermatitis increased, indicating an increased number of patients diagnosed with atopic dermatitis who received healthcare in 2019. Healthcare for atopic dermatitis patients during 2020 in Colombia suffered the impact of COVID-19, causing a decrease in the medical care of patients diagnosed with atopic dermatitis.

This work sets the grounds for future research with other methodologies that will allow a more accurate calculation of the prevalence of atopic dermatitis in Colombia. The present study can be helpful for physicians to gain a better understanding of the disease and improve atopic dermatitis patient management.

## Supplementary files

**Supplementary table 1.** Distribution of the indicated comorbidities among patients using healthcare related to a primary atopic dermatitis diagnosis according to an ICD-10 chapter by year

ICD-10 chapter	2015			2016		2017		2018		2019	
	n	%*	n	%*	n	%*	n	%*	n	%*
I-Certain infectious and parasitic diseases	4,294	1.3	3,241	1.5	4,776	2.1	5,056	2.0	4,541	1.5
II-Neoplasms	174	0.1	200	0.1	607	0.3	641	0.3	319	0.1
III-Diseases of the blood and blood-forming organs and certain disorders involving the immune mechanism	309	0.1	279	0.1	469	0.2	392	0.2	300	0.1
IV-Endocrine, nutritional, and metabolic diseases	1,732	0.5	1,608	0.8	2,419	1.1	3,180	1.3	2,179	0.7
V-Mental and behavioral disorders	335	0.1	374	0.2	569	0.3	610	0.2	554	0.2
VI-Diseases of the nervous system	272	0.1	345	0.2	374	0.2	456	0.2	402	0.1
VII-Diseases of the eye and adnexa	1,208	0.4	1,023	0.5	1,840	0.8	1,524	0.6	1,199	0.4
VIII-Diseases of the ear and mastoid process	389	0.1	478	0.2	486	0.2	532	0.2	479	0.2
IX-Diseases of the circulatory system	954	0.3	997	0.5	3,260	1.4	2,152	0.9	1,095	0.4
X-Diseases of the respiratory system	2,630	0.8	2,752	1.3	4,028	1.8	4,229	1.7	3,043	1.0
XI-Diseases of the digestive system	1,325	0.4	1,578	0.7	1,857	0.8	1,846	0.7	1,636	0.6
XII-Diseases of the skin and subcutaneous tissue	265,544	83.2	173,844	82.1	183,890	80.8	198,835	78.8	249,364	84.3
XIII-Diseases of the musculoskeletal system and connective tissue	1,794	0.6	1,862	0.9	2,127	0.9	2,239	0.9	1,884	0.6
XIV-Diseases of the genitourinary system	1,421	0.4	1,363	0.6	1,762	0.8	2,479	1.0	1,330	0.4
XVIII-Symptoms, signs and abnormal clinical and laboratory findings, not elsewhere classified	3,126	1.0	2,505	1.2	3,731	1.6	4,600	1.8	2,994	1.0
XXI-Factors influencing health status and contact with health services	43,327	13.6	27,857	13.2	31,752	14.0	38,088	15.1	35,877	12.1

* Proportion calculated with the number of patients with a secondary diagnosis of a specific chapter of the ICD-10 in a year over the number of patients with any given concomitant diagnosis in that year

**Supplementary table 2.** Number of patients with comorbidities receiving healthcare attention related to a primary atopic dermatitis diagnosis by year*

Age group (years)	2015	2016	2017	2018	2019
0-11	109,045	82,678	94,375	104,559	105,518
12-17	25,216	18,335	21,394	24,460	26,315
18-29	48,775	32,412	36,509	39,415	49,139
30-59	98,373	54,632	53,832	58,398	80,063
>60	37,729	23,752	21,407	25,412	34,674
Total	319,069	211,809	227,517	252,244	295,709

**Supplementary table 3.** Number of patients with a registered prescription of dupilumab and dupilumab syringes prescribed between 2015 and 2020 by the professional in charge of the prescription

Specialty	2019		2020	
	Patients	Prescribed syringes	Patients	Prescribed syringes
Dermatologist	363	5,811	614	11,870
Allergist	157	2,784	236	4,313
Primary physician	59	718	133	2,047
Pediatrician	16	392	18	399
Master's degree	12	127	17	321
Internal medicine physician	3	38	8	182
Family physician	-	-	4	44
Oncological dermatology	1	4	2	34
Other clinical specialties	2	30	-	-
PhD degree	-	-	1	22
Otology	1	16	-	-
Family and community health	1	8	-	-
Other multidisciplinary specialization	-	-	1	8
Total	569	9,928	928	19,240
